# High-dose Vitamin D supplementation for immune recalibration in autoimmune diseases

**DOI:** 10.3389/fimmu.2025.1625769

**Published:** 2025-08-12

**Authors:** Shiuan-Tzuen Su, Po-Cheng Shih, Meng-Che Wu

**Affiliations:** ^1^ Department of Surgery, Chung Shan Medical University Hospital, Taichung, Taiwan; ^2^ School of Medicine, Chung Shan Medical University, Taichung, Taiwan; ^3^ Institute of Medicine, Chung Shan Medical University, Taichung, Taiwan; ^4^ Division of Allergy, Immunology and Rheumatology, Department of Internal Medicine, Changhua Christian Hospital, Changhua, Taiwan; ^5^ Division of Pediatric Gastroenterology, Children’s Medical Center, Taichung Veterans General Hospital, Taichung, Taiwan; ^6^ Department of Post-Baccalaureate Medicine, College of Medicine, National Chung Hsing University, Taichung, Taiwan

**Keywords:** high-dose, vitamin D, immune, autoimmune disease, VDR

## 1 Autoimmune disease pathophysiology and immune dysregulation

Hileman et al. reported that ADs arise from a complex interplay of genetic susceptibility and environmental triggers, with viral infections playing a central role (1). Viruses activate innate immunity by inducing type I interferon (IFN-α/β) production and stimulating neutrophil extracellular trap (NET) release, thereby enhancing dendritic cell maturation and antigen presentation. In genetically predisposed individuals, these events promote adaptive immune activation, leading to B- and T-cell expansion, autoantibody generation, and T-cell dysregulation.

Robinson et al. further proposed that Epstein-Barr virus (EBV) contributes to autoimmunity through multiple mechanisms: molecular mimicry of human autoantigens, reprogramming of B-cell function, and binding of EBV nuclear antigen 2 (EBNA2) to host super-enhancer regions associated with autoimmune-susceptibility genes (2). Together, these actions disrupt normal gene regulation and perpetuate chronic immune activation.

Taken together, these studies underscore the pivotal role of viral infections in both initiating and exacerbating ADs and highlight potential antiviral or immunomodulatory targets for therapeutic intervention.

## 2 Vitamin D deficiency in autoimmune diseases and its immunomodulatory potential

According to the recent estimation, nearly 15 million Americans live with at least one ADs. Increasing evidence suggests that vitamin D insufficiency is highly prevalent in these patients and correlates with immune dysregulation, higher disease activity, and more frequent flares (3). Vitamin D supplementation was important to prevent and treat deficiency-related conditions like rickets. However, in vitamin D-replete adults, large-scale randomized trials and Mendelian randomization studies consistently showed no significant benefit for preventing cancer, cardiovascular disease, diabetes, or fractures. High-dose supplementation may even pose risks. Thus, routine use in the general population is not supported, except to correct deficiency or in specific at-risk groups (4).

Epidemiological studies have linked low serum 25-hydroxyvitamin D [25(OH)D] levels (<20 ng/mL) to elevated risk for ADs such as psoriasis, T1D, and multiple sclerosis (MS) (5). Vitamin D contributes to immune homeostasis by promoting innate defenses, enhancing macrophage and dendritic cell function, while modulating adaptive responses through suppression of Th1- and Th17-mediated inflammation and upregulation of regulatory T cells (6).

In MS specifically, vitamin D influences lymphocyte activation, T-helper cell polarization, and cytokine production. It decreases pro-inflammatory cytokines (e.g., IFN-γ, IL-17) and increases anti-inflammatory mediators (e.g., IL-10), thereby shifting the immune milieu toward tolerance (7). Randomized trials of supplementation (e.g., 4,000 IU/day cholecalciferol) have demonstrated significant reductions in relapse rates and Magnetic Resonance Imaging (MRI) lesion burden in relapsing–remitting MS, particularly in patients with baseline 25(OH)D <30 ng/mL (8).

Taken together, these findings support a therapeutic role for vitamin D in ADs, especially MS, by rebalancing innate and adaptive immunity and modulating key cytokines such as IL-10 and IL-17. Future large-scale trials are warranted to define optimal dosing, target serum levels, and long-term safety profiles.

## 3 Relevance to vitamin D receptor signaling and immune regulation (T-cell modulation and cytokine suppression)

The hormonally active metabolite of vitamin D, 1,25-dihydroxyvitamin D3 [1,25(OH)2D3], exerts its immunomodulatory functions primarily by binding the vitamin D receptor (VDR), a nuclear transcription factor expressed across diverse immune cell types (5). Upon ligand engagement, VDR heterodimerizes with retinoid X receptor and associates with vitamin D response elements in target gene promoters, thereby regulating transcription. This VDR-mediated gene regulation underlies vitamin D’s capacity to shape both innate and adaptive immune responses.

In the innate arm, 1,25(OH)2D3–VDR signaling promotes monocyte-to-macrophage differentiation, enhances expression of antimicrobial peptides such as cathelicidin, and upregulates HLA-DR and co-stimulatory molecules on dendritic cells, facilitating more effective antigen presentation (5). Concomitantly, VDR activation modulates cytokine and chemokine profiles within the innate compartment, fostering an environment that supports pathogen clearance while limiting excessive inflammation.

**Figure 1:**
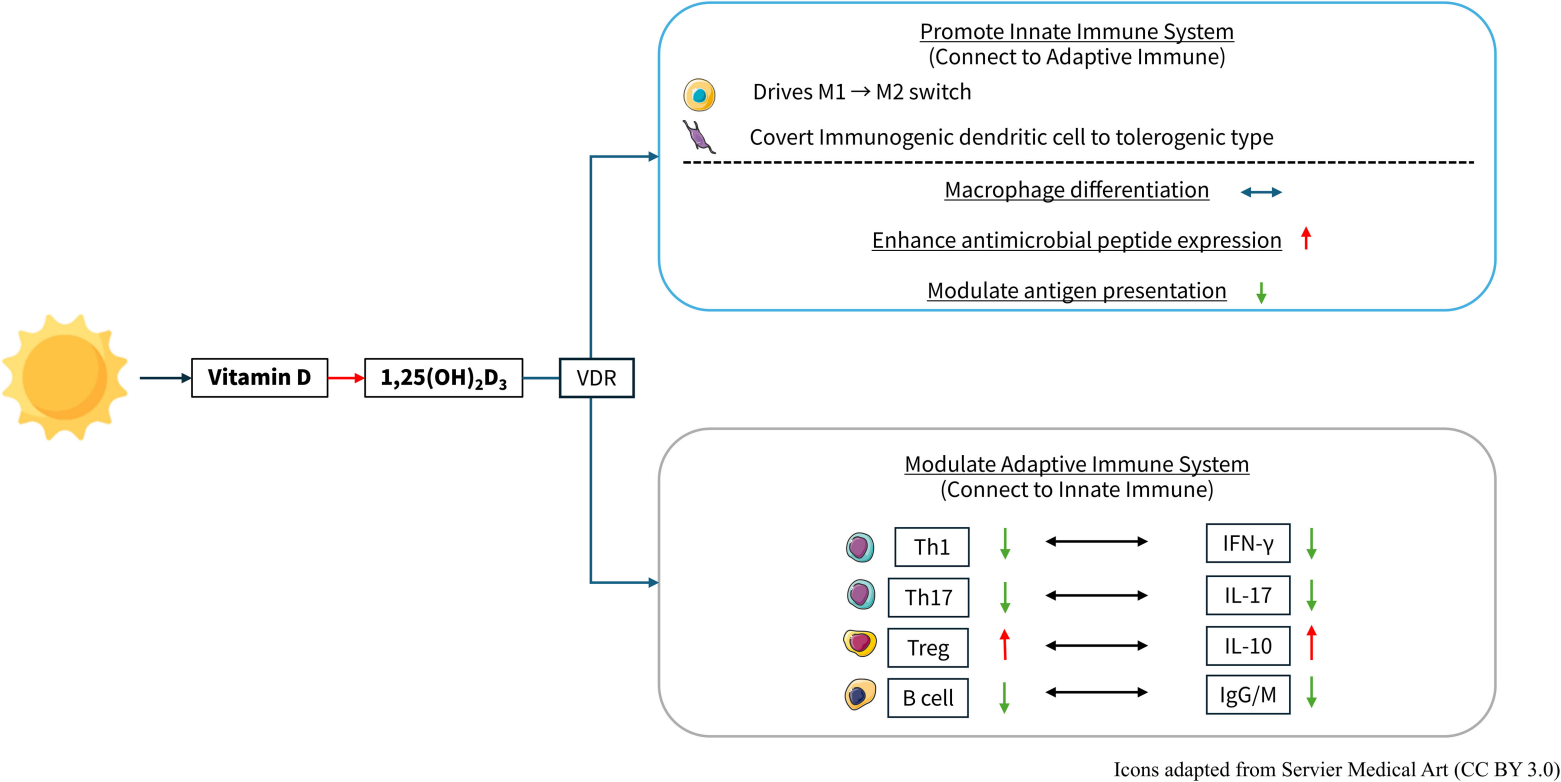
The active form of vitamin D, 1,25-dihydroxyvitamin D_3_, exerts immunomodulatory effects on both the innate and adaptive immune systems. Vitamin D and Immune Regulation. 1,25-(OH)_2_D_3_: vitamin D, 1,25-dihydroxyvitamin D_3_; VDR, Vitamin D receptor; Th, T helper cell; IFN-γ, interferon-γ; IL, interleukin; Treg, regulatory T cells; IgG, immunoglobulin G; IgM, immunoglobulin M. https://smart.servier.com/.

Within adaptive immunity, VDR signaling exerts a potent suppressive effect on Th1- and Th17-mediated inflammation by directly downregulating transcription of IFN-γ and IL-17, respectively. At the same time, it promotes the expansion and functional stability of regulatory T cells (Tregs), enhancing IL-10 production and strengthening peripheral tolerance. (5, 9) B-cell activity is similarly restrained: VDR activation inhibits plasma cell differentiation and autoantibody secretion, thereby reducing humoral autoimmunity (10). **Figure 1** revealed the relationship between vitamin D and immune modulation.

Beyond direct effects on immune cells, Sîrbe et al. (5) have reported that VDR signaling contributes to the maintenance of gut barrier integrity and the modulation of microbiota composition, mechanisms increasingly implicated in the pathogenesis of ADs. Clinical investigations echo these molecular insights. Zhao et al. (11) and Manousaki et al. (12) observed that vitamin D supplementation was associated with lower incidence rates and reduced disease severity in SLE and T1D cohorts. Taken together, these findings highlight VDR-dependent pathways as promising targets for therapeutic strategies aiming to recalibrate immune homeostasis in autoimmune disorders.

## 4 The efficacy and safety of high-dose vitamin D supplementation in modulating immune profiles in autoimmune disease

High-dose cholecalciferol is increasingly investigated to overcome VDR sensitivity loss in genetically predisposed individuals, where pathogen-mediated VDR downregulation, chronic glucocorticoid exposure, environmental toxins, low UVB exposure, and aging all contribute to impaired vitamin D signaling and elevated autoimmune risk (13). However, the efficacy of high-dose vitamin D remains debated (14–19). **Table 1** summarizes clinical trials of high-dose vitamin D in autoimmune diseases.

**Table 1:** Summary of high dose vitamin D for autoimmune disease.

Author	Participants	Vitamin D dose	Disease	Age	Sex	Study location	Results	Study design	Level of evidence
Cassard S.D. et al. (14)	172	600 IU/day (LDVD) or 5000 IU/ day (HDVD)	Relapsing- remitting multiple sclerosis	18–50 years	Not specified	16 neurology clinics in the US	HDVD did not reduce disease activity	RCT	Level I
Aranow C. et al. (15)	57	2,000 or 4,000 IU/day	Systemic lupus erythematosus	36–39 years	Female 94.4%	8 centers in US	Failed to diminish the IFN signature in vitamin D–deficient Systemic lupus erythematosus	RCT	Level I
Grove-Laugesen D. et al. (16)	278	2800 IU/day	Graves’ Disease	44 ± 14 years	Female 79%	7 endocrine clinics, Denmark	High-dose vitamin D does not recommend for Graves’ Disease	RCT	Level I
Nwosu B.U. et al. (17)	36	50000 IU/ week (2 months), then Q2W (10 months)	Type 1 diabetes	10–21 years	Not specified	Not specified	Partial clinical remission	RCT	Level I
Finamor D.C. et al. (18)	9 psoriasis and 16 vitiligo	vitamin D3 35,000 IU/day for 6 months	Psoriasis and vitiligo	47.8 years old	Female 28%	2 clinics, Brazil	Effective and safe for vitiligo and psoriasis patients	Retrospective	Level II
Fernandes A.L. et al. (19)	144	vitamin D3 200,000 IU	moderate to severe COVID-19	54.3 years old	Female 46.5%	São Paulo,and Ibirapuera Field Hospital, Brazil	not support symptoms control	RCT	Level I
Thouvenot E. et al. (21)	316	oral cholecalciferol 100–000 IU/2 weeks for 24 months.	Isolated syndrome and early relapsing remitting multiple sclerosis	18–55 years	Female 69.6%	36 Multiple sclerosis centers, France	Reduced disease activity in Isolated syndrome and early relapsing remitting multiple sclerosis	RCT	Level I
Bendix M. et al. (22)	40	20,000 IU/day for seven weeks and infliximab infusion with 5 mg/kg	Crohn’s Disease	Not specified	Not specified	Aarhus University Hospital	Reduced infliximab dose escalation	RCT	Level I

(LDVD), Low dose vitamin D3; (HDVD), high dose vitamin D3; RCT, Randomized Controlled Trial; US, United States; IFN, interferon; Q2W, every other week.

Several studies have revealed high-dose vitamin D_3_ supplementation in autoimmune diseases but reported no significant clinical benefits. However, many of these studies have important methodological limitations that may affect the interpretation of their findings. For instance, the study by Cassard SD et al. (14) involved a relatively small sample size, lacked a placebo control, and was conducted only in the United States, limiting its generalizability. Similarly, the trial by Aranow C et al. (15) showed patients with SLE, was also restricted by a small participant pool, a short follow-up period of only 12 weeks, and a predominantly female population with varied baseline disease activity, introducing potential heterogeneity in treatment response. The study by Grove-Laugesen D et al. (16) was also limited to a single country (Denmark), and included mostly female participants, raising concerns about gender representation and external validity. In the trial by Nwosu BU. et al. (17), the limitations included a small sample size, a narrow age range, a single-country of Denmark. Lastly, the study conducted by Fernandes AL et al. (19) assessed the effects of a single high-dose intervention (200,000 IU) and faced challenges such as a limited sample size for 1 year analysis and reliance on self-reported symptoms, which may compromise the reliability of the outcome assessment.

Brustad et al. (20) conducted a systematic review and meta-analysis of 32 randomized trials (n = 8,400 children, doses 1,200–10,000 IU/day; bolus up to 600,000 IU) and found no increase in serious adverse events, including hypercalcemia or nephrolithiasis. In France, Thouvenot et al. (21) treated 316 early MS patients with 100,000 IU cholecalciferol biweekly for 24 months, observing reduced relapse rates and MRI lesion accumulation in clinically isolated syndrome and relapsing–remitting MS. Bendix et al. (22) administered 20,000 IU/day for seven weeks to 40 Crohn’s disease patients, reporting a 25% decrease in the need for infliximab dose escalation.

These data suggest that high-dose vitamin D can safely modulate immune profiles, decrease disease activity, and potentiate existing therapies in autoimmune disorders. Nonetheless, optimal dosing regimens, especially for individuals with profound deficiency or specific disease phenotypes, require further large randomized trials to balance maximal immunomodulation against potential toxicity.

## 5 Future research and study design

Future research should focus on well designed, randomized controlled trials that enroll patients based on their baseline 25(OH)D levels, VDR related genetic variants, and specific autoimmune phenotypes. Such trials ought to include dose finding phases to identify effective yet safe vitamin D regimens, serial immunological assessments (e.g., Th1/Th17 cytokines, Treg frequencies, autoantibody titers), and disease specific clinical endpoints (relapse rates, imaging markers, or activity indices). Close monitoring for hypercalcemia and renal effects will ensure safety, while stratified analyses will reveal which patient subgroups derive the greatest benefit from high dose supplementation.

## 6 Conclusion

Vitamin D is essential for immune balance, and its deficiency contributes to autoimmunity. High-dose vitamin D can rebalance Th1/Th17 versus Treg activity, lessen disease flares, and boost standard therapies without raising serious safety concerns. Tailoring supplementation to patients’ baseline levels and genetics offers a promising adjunct in managing autoimmune diseases.
